# The Recovery of Muscle Spindle Sensitivity Following Stretching Is Promoted by Isometric but Not by Dynamic Muscle Contractions

**DOI:** 10.3389/fphys.2020.00905

**Published:** 2020-08-04

**Authors:** Francesco Budini, Dietmar Rafolt, Monica Christova, Eugen Gallasch, Markus Tilp

**Affiliations:** ^1^Institute for Sport Science, Graz University, Graz, Austria; ^2^Center for Medical Physics and Biomedical Engineering, Medical University of Vienna, Vienna, Austria; ^3^Otto Loewi Research Center, Physiology Section, Medical University of Graz, Graz, Austria; ^4^Institute of Physiotherapy, Institute of Applied Sciences FH-Joanneum, Graz, Austria

**Keywords:** static stretching, tap reflex, muscle spindle, thixotrophy, dynamic contractions

## Abstract

It is often suggested that stretching-related changes in performance can be partially attributed to stretching-induced neural alterations. Recent evidence though shows that neither spinal nor cortico-spinal excitability are susceptible of a long-lasting effect and only the amplitude of stretch or tap reflex (TR) is reduced up to several minutes. Since afferents from muscle spindles contribute to voluntary muscle contractions, muscle stretching could be detrimental to muscle performance. However, the inhibition of muscle spindle sensitivity should be reversed as soon as the stretched muscle contracts again, due to α-γ co-activation. The present work evaluated which type of muscle contraction (static or dynamic) promotes the best recovery from the inhibition in spindle sensitivity following static stretching. Fifteen students were tested for TR at baseline and after 30 s maximal individual static stretching of the ankle plantar flexors followed by one of three randomized interventions (isometric plantar flexor MVC, three counter movement jumps, and no contraction/control). Ten TRs before and 20 after the procedures were induced with intervals of 30 s up to 10 min after static stretching. The size of the evoked TRs (peak to peak amplitude of the EMG signal) following stretching without a subsequent contraction (control) was on average reduced by 20% throughout the 10 min following the intervention and did not show a recovery trend. Significant decrease in relation to baseline were observed at 9 of the 20 time points measured. After MVC of plantar flexors, TR recovered immediately showing no differences with baseline at none of the investigated time points. Following three counter movement jumps it was observed a significant 34.4% group average inhibition (*p* < 0.0001) at the first time point. This effect persisted for most of the participants for the next measurement (60 s after intervention) with an average reduction of 23.4% (*p* = 0.008). At the third measurement, 90 s after the procedure, the reflexes were on average still 21.4% smaller than baseline, although significant level was not reached (*p* = 0.053). From 120 s following the intervention, the reflex was fully recovered. This study suggests that not every type of muscle contraction promotes a prompt recovery of a stretch-induced inhibition of muscle spindle sensitivity.

## Introduction

It is often suggested that stretching-related changes in performance (Behm et al., 2001; Bradley et al., 2007) can be partially attributed to stretching-induced neural alterations (Kay and Blazevich, 2009), however, the magnitude and the duration of these neural effects do not seem to support these assumptions. Indeed, although it has been extensively reported that muscle stretching induces several neuromuscular responses (For review Budini and Tilp, 2016), H-reflex and motor evoked potential return to baseline values within seconds (Guissard et al., 2001; Budini et al., 2018a), whilst only the amplitude of tap or stretch reflex seems to be susceptible of a long lasting effect (Avela et al., 1999; Budini et al., 2017, 2019). However, this inhibition cannot be attributed to changes in the reflex loop, but rather to a thixotropy effect within the muscle spindles that reduces their sensitivity with a consequent decrease of Ia afferents activity (Proske et al., 1993). Therefore, this long-lasting inhibition seems to have a mechano-morphological rather than a neural nature. Regardless of its nature, a decreased muscle spindle sensitivity does have neural implications. It has long been known, in fact, that afferents from muscle spindles contribute in various ways to different voluntary muscle contractions (Vallbo, 1974; Burke et al., 1978; Macefield et al., 1991). A paper by Dietz et al. (1979) showed for example a decreased presynaptic inhibition of Ia afferent during running. Another study by Meunier and Morin (1989) demonstrated that both homonymous and heteronymous Ia facilitations are markedly increased at the beginning of a voluntary isometric contraction (Meunier and Morin, 1989). The contribution of spindles afferents to contraction is also in relation to agonist heteronymous decrease of presynaptic inhibition on Ia homonymous afferents, with simultaneous increase of presynaptic inhibition of Ia in the antagonist (Hultborn et al., 1987). Voluntary contractions are also supported by muscle spindles through the γ loop (Hagbarth et al., 1986). Consequently, muscle stretching could be detrimental to muscle performance. Indeed several studies reported a decrease in maximal static and dynamic force following stretching (for review Behm and Chaouachi, 2011) and in this respect a decreased contribution of spindle afferents to the contraction could be imputed as one of the possible underlying mechanisms.

However, the relevance to sport practice of these studies is limited because most research works have adopted stretching protocols of over 30 s duration (Rosenbaum and Hennig, 1995; Weir et al., 2005) or have repeated the intervention to the same muscle several times (Avela et al., 1999, 2004). These procedures are very different to standard stretching practice in sport. Another element that cannot be fully elucidated and that would be relevant to sport performance is the duration of the effect. It is known that even short duration stretching bouts (2 times 30 s) inhibit tap reflex for several seconds (Budini et al., 2017), but the time course of the recovery has only been investigated within the first 30 s showing a very slow recovery slope (Budini et al., 2018a). Other studies reported a full recovery within 5–10 min following the stretching procedure (Budini et al., 2017, 2019), but this cannot be considered conclusive since the volunteers were stretched and tested when sitting, but stood up after 5 min for some rest before sitting again for continuing the measurements. Since standing up requires the voluntary activation of the leg muscles, it is reasonable to assume that the slack in muscle spindles induced by the stretching procedure was regained with the voluntary contraction of the stretched muscle with related α-γ co-activation. In conclusion, to date it is not clear for how long muscle spindle sensitivity is reduced following a stretching exercise like those adopted commonly in sport practise (around 30 s static stretching per muscle group). Moreover, it is not known whether a voluntary muscle contraction is sufficient to fully restore muscle spindle sensitivity.

We can anticipate that a maximal voluntary contraction (MVC) of the stretched muscles would re-establish promptly muscle spindle sensitivity via α-γ co-activation, however, such task does not represent a standard routine that can be easily implemented within a warm up session before a performance, therefore this expected result would limit its practical application. On the other hand, dynamic contractions, in the form of counter movement jumps (CMJs) are already commonly adopted in warm up routines, and also in this case we could expect a similar reflex recovery based on the same mechanism. However, both these assumptions have to be examined.

Therefore, in the present work we investigate the effects of a single 30 s triceps surae stretching exercise on tap reflex inhibition and its recovery during the following 10 min in three different conditions: with an MVC following stretching, with three CMJs following stretching and without any contraction.

## Materials and Methods

### Participants

Fifteen recreationally active students (7 male age 25.0 ± 2.8 years. body mass 80.1 ± 9.9 kg, stature 184.9 ± 5.4 cm, and 8 female age 24.6 ± 3.1 years, body mass 61.3 ± 8.8 kg, stature 168.6 ± 5.9 cm) with no history of neurological disorders volunteered for the experiment. Volunteers were required to abstain from any strenuous physical activity before the testing day and, due to the known effects of nicotine (Ginzel et al., 1969) and caffeine (Walton et al., 2003) on spinal reflexes excitability, were also asked to refrain from taking caffeine-containing substances and smoking within 2 h before the testing session. The study was approved by the local research ethics board and written informed consent was obtained from all volunteers before the onset of the experimental procedures.

### Study Design

Participants attended the laboratory on a single occasion lasting about 90 min. The experiment consisted of measuring the tap reflex before and after stretching of ankle plantar flexors with three different interventions (isometric contraction, dynamic contraction and no contraction) in between the before and after stretching assessments.

### Experimental Procedures

Subjects were sitting on an isokinetic dynamometer (CON-TREX MJ, CMV AG, Duebendorf, Switzerland) with the standard setup for ankle joint movement individually adjusted. Participants had their right knee fully extended and the foot resting on the dynamometer footplate, the ankle joint aligned with the dynamometer rotation shaft and the ankle angle set at neutral position of 90°. Volunteers sat with the trunk at 110° and the head supported by a polystyrene ball filled cushion (dentafix^®^, pro medico HandelsGmbH, Graz, Austria) that once positioned could be deflated thanks to a specially designed valve allowing the formation of a stable form molded on the volunteers’ head and neck shapes. In this arrangement the backside and the heel of the tested foot were close fitting against fixed ends (seatback and dynamometer footplate), consequently, being the knee fully extended, any slide in the sitting position was not possible. This placement allowed us to have the volunteer to stand up from the dynamometer chair and come back to the same testing position. By using a remote control, the volunteers were instructed to adjust the dorsiflexion isokinetic rotation operated by the dynamometer around the foot plate until the point of perceived maximal dorsiflexion. Participants were asked to keep their knee extended and to relax during the procedures.

Once the maximal individual dorsiflexion was defined, subjects left the dynamometer and were prepared for electromyographic recording (EMG) from soleus (SOL) and gastrocnemius medialis (GM) muscles. Subsequently, the volunteers sat down again on the dynamometer chair (position described above) and were then instructed to relax completely and gaze horizontally at a point set 4 m distant. After 30 s sitting in testing position the first tap reflex was evoked, further nine reflexes were induced with intervals of 30 s.

**FIGURE 1:**
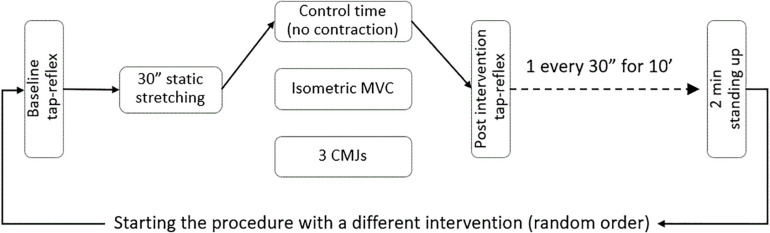
Experimental flow chart.

Following baseline recordings, the foot was passively rotated (5°/s) to the individual maximal dorsiflexion. This position was kept for 30 s and then the foot was passively rotated (5°/s) back to 90°. Before the first tap reflex post-stretch was evoked, one of three interventions was introduced: control, 3 s maximal isometric contraction of the stretched plantar flexors, or three counter movement jumps. Thirty seconds after the intervention, the collection of tap reflexes started again and continued for 10 min with one reflex elicited every 30 s; in this way it was possible to collect data at 20 different time points. The complete procedure was repeated a second and third time so to complete the remaining interventions. Between one complete pre-post stretch measurement and the next, the volunteers were asked to stand up for 2 min. The order of the interventions was randomized. A flow chart of the experimental sequence is presented in Figure 1.

### Surface Electromyography

Volunteers were prepared for surface EMG from the SOL and GM of the right leg. After appropriate skin preparation, electrodes (Blue Sensor N, Ambu A/S, Ballerup, Denmark) were placed in monopolar configuration and the ground electrode was placed over the tibial bone medial surface.

### Tap Reflex

Tendon tap reflex was elicited by a hammer driven by motor (Type GDRX 075, Magnet-Schultz, Germany) hitting the Achilles tendon about 3–4 cm above its insertion on the calcaneus. An electrical output from the motor provided information about its rotation allowing hammer hitting consistency to be monitored.

### Data Analysis

Electromyography, foot displacement, torque and trigger signals were synchronized (Dewetron 7.0 recording system), digitized with a sampling frequency of 5 KHz, stored on a PC and analyzed using custom algorithms developed in Matlab (R2014b).

Tap reflexes were estimated as reflex peak to peak amplitude of the raw EMG signal. Peak torque was evaluated as the peak in the torque response produced by the reflex. All data were checked for consistency and values exceeding ±2 standard deviations within their own recording series (i.e., within a subject) were discarded (31 values) (Budini et al., 2019); all the remaining values (801 values) were retained and used for statistical analysis.

### Statistical Analysis

Mean values and standard deviations of tap reflexes and peak torque data are reported. Sex differences at baseline before stretching were checked by 2-sample *t*-test.

Repeated measures ANOVA was applied to compare the three baselines before the interventions (Control, MVC, and CMJ).

Comparisons between baseline values versus each of the 20 time points following the interventions were performed with Dunnett’s test for both tap reflex EMG and hammer motor output. To account for the great individual variability of the reflex values, the individual mean values of all 21 measures within the subjects were subtracted from each value.

To compare the effects of the three interventions, a two-way repeated measures ANOVA (factors: group, time, interaction) was applied on the delta variations from baseline. *Post hoc* repeated measures ANOVAs were then applied to determine differences at specific time points.

All statistical analysis was completed using PASW Statistic 18.0.0.

## Results

The analysis of the hammer motor output confirmed the consistency in tendon hits: no differences were observed between average baseline value and any of the post intervention time points (*p* = 1.000 for every comparison within each of the three conditions).

Average tap reflex wave amplitude and standard deviation for SOL was 1.09 ± 0.70 mv for females and 1.21 ± 1.05 mv for males at baseline control; 1.06 ± 0.76 mv for females and 1.30 ± 0.95 mv for males at baseline MVC; and 1.08 ± 0.71 mv for females and 1.12 ± 0.81 mv for males at baseline CMJ. No differences between genders were observed at neither of these (*p* = 0.80, *p* = 0.60, and *p* = 0.92, respectively), consequently data were collapsed into a single group. Repeated measure ANOVA performed on the data of collapsed groups showed no differences at baseline between the three conditions (*p* = 0.535, *F* = 0.64).

Similarly, twitch torque was not different between genders at none of the three baseline measurements (*p* = 0.25, *p* = 0.15, and *p* = 0.31 for control, MVC and CMJ, respectively). Group average values and standard deviations at baselines were 5.6 ± 2.9, 6.2 ± 3.3, and 5.9 ± 3.1 Nm for control, MVC and CMJ, respectively, and not significantly different between each other (*p* = 0.97, *F* = 0.029).

**FIGURE 2:**
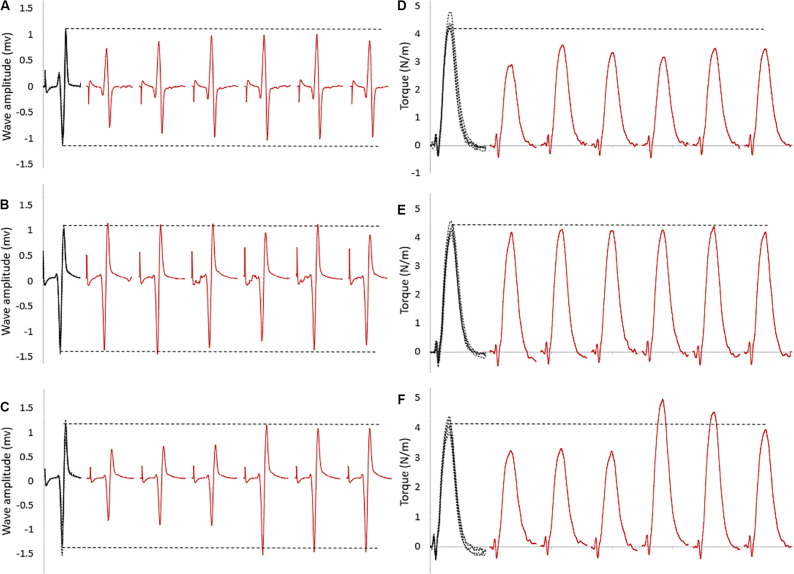
Raw SOL EMG data of tap reflexes: in black at baseline (five superimposed tracks); in red the first six tap reflexes (from 30 to 180 s) following stretching combined with **(A)** no contraction, **(B)** MVC, **(C)** CMJs. The horizontal dashed lines represent the baseline average values. The corresponding twitch torque values are pictured in **(D–F)**.

For within intervention analysis, Figure 2 shows a sample of raw data recording at baseline and subsequent six stimulation points. As visible in this representative data, the size of the evoked reflexes remained inhibited following stretching in the control intervention (no contraction, Figure 2A), returned immediately to baseline level when stretching was followed by an MVC (Figure 2B) and remained inhibited for the first three stimulations for the then recover after the stretching and CMJ conditioning intervention (Figure 2C).

**FIGURE 3:**
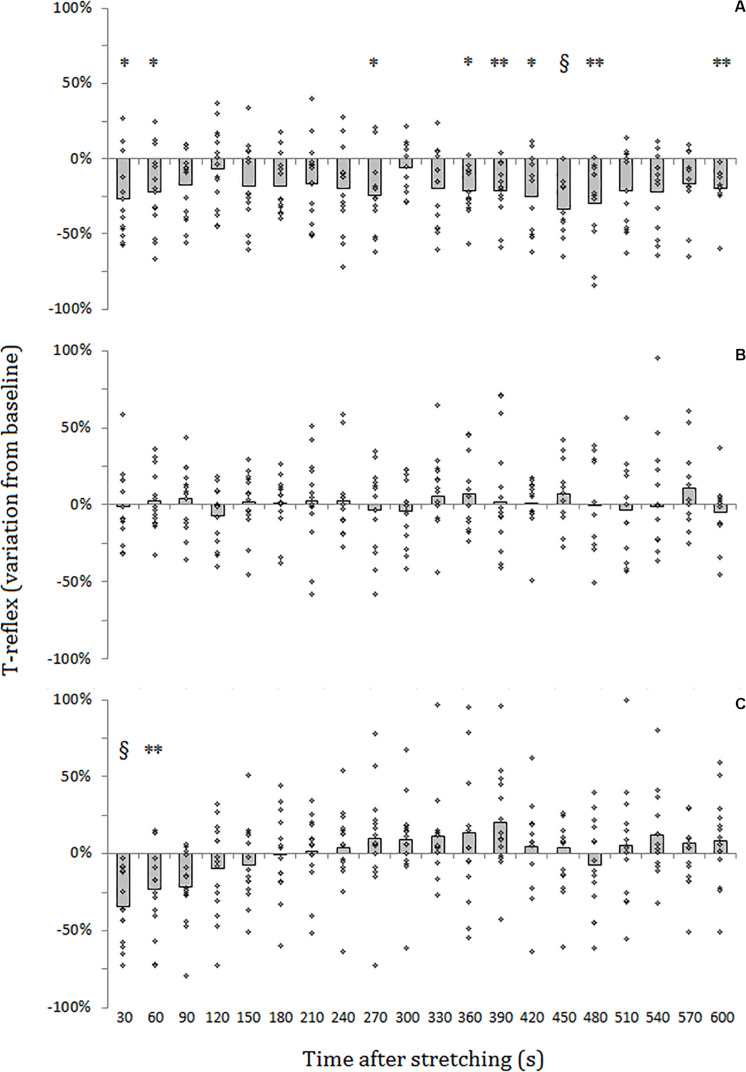
Percentage variation from baseline of SOL tap reflex amplitude for group average (gray bars) and individual values (dots). **(A)** No contraction after stretching, **(B)** maximal isometric contraction after stretching, **(C)** dynamic contraction after stretching. ^§^ = *p* < 0.001; ** = *p* < 0.01; * = *p* < 0.05.

**FIGURE 4:**
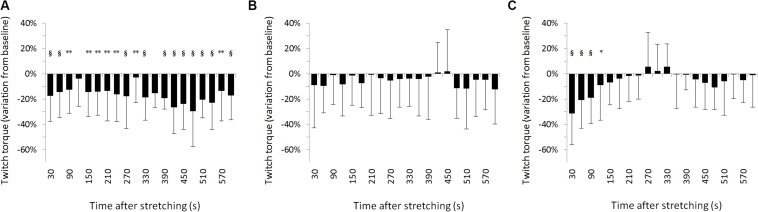
Percentage variation from baseline of twitch torque for group average. **(A)** No contraction after stretching, **(B)** maximal isometric contraction after stretching, **(C)** dynamic contraction after stretching. ^§^ = *p* < 0.001; ** = *p* < 0.01; * = *p* < 0.05.

For SOL muscle, group average results resemble those presented in the representative plot in Figure 2: the size of the evoked tap reflexes following stretching without a subsequent contraction was on average smaller at every time point after stretching, although significant levels were reached only at 9 out of 20 time points (30, 60, 270, 360, 390, 420, 450, 480, and 600 s) following stretching. The average reduction was 20% ranging from 5.9% (recorded 300 s after stretching) and 33.5% (420 s after stretching) (Figure 3A). The tap reflex did not show a recovery trend. For GM muscle, despite an overall similar behavior, significant differences were observed only 480 s after stretching (*p* = 0.038). Similarly, the twitch torque was decreased at every time point and showed a group average reduction of 16.6% from baseline (sample of recordings in Figure 2D). Significant levels were reached at every but one time point (120 s, *p* = 0.085) following stretching (Figure 4A).

When stretching was followed by a maximal voluntary contraction of plantar flexors, tap reflex recovered immediately, showing no differences with baseline at any of the investigated time points (Figure 3B). Same result was also observed for GM muscle. Group average twitch torque values were lower than baseline at 18 of the 20 time points (average variation −5% ranging from −12% to +2%) (Figure 4B), however, the Dunnet’s test revealed no significant differences at any time point following the procedure, compared to baseline.

Thirty seconds following the intervention stretching-CMJ a marked decrease in reflex size was observed in all the subjects with the reflex amplitude dropping from a group average of 1.10 ± 0.73 to 0.78 ± 0.67 mv (28.7% group average inhibition in comparison to baseline) (*p* < 0.0001). This effect persisted for most of the participants for the next measurement with an average reduction of 22.0% (from 1.10 ± 0.73 to 0.86 ± 0.69 mv) (*p* = 0.008). At the third measurement following the intervention (after 90 s) a 18.1% group average reduction in reflex size compared to baseline (from 1.10 ± 0.73 to 0.90 ± 0.76 mv) could still be observed, although significant level was not reached (*p* = 0.053). Tap reflex recovered within 120 s (1.03 ± 0.8 mv) and no other differences from baseline were observed (Figure 3C). Data from GM muscle are similar to those reported for SOL: significant reductions were observed at the first and second time points after the procedure (*p* = 0.001 and *p* = 0.003, respectively).

Twitch torque values got in line with tap reflex results: a group average inhibition of 35.6% was observed at the first measurement (from 5.9 ± 3.1 to 3.8 ± 0.11 Nm) (*p* < 0.0001), 20.9% inhibition at the second (from 5.9 ± 3.1 to 4.6 ± 0.12 Nm) (*p* < 0.0001), and 18.8% inhibition at the third (from 5.9 ± 3.1 to 4.8 ± 0.13 Nm) (*p* < 0.0001) measurement. A significant reduction from baseline was observed also for the fourth measurement 120 s following stretching (11.8% inhibition, from 5.9 ± 3.1 to 5.2 ± 0.12 Nm, *p* = 0.04343) (Figure 4C). There was no significant difference onward from 150 s following stretching.

The two-way repeated measures ANOVA, applied on the delta variations from baseline to compare the effects of the three interventions, revealed a group^∗^time effect (*p* = 0.046, *F* = 1.74). Comparisons at the same time points between conditions highlighted differences at 30 and 60 s after intervention (*p* = 0.013, *F* = 5.75, and *p* = 0.005, *F* = 6.66, respectively). Pairwise analysis showed, for 30 s after intervention, that the inhibition of the reflex after CMJ was different in comparison to MVC (*p* = 0.008), but did not reach significant level in comparison to control (*p* = 0.08). At 60 s after intervention, the inhibition of the reflex after CMJ was different in relation to both MVC (*p* = 0.006) and control (*p* = 0.003). At the third time point (*p* = 0.0014, *F* = 5.08) MVC resulted different from both control (*p* = 0.029) and CMJ (*p* = 0.029).

Other significant differences were found at time points 210, 240, 270, 300, 420, 450, and 600 s after intervention. In all these cases control condition resulted different from CMJ, MVC or both and in three cases (at time points 240, 300, and 390) pairwise comparison also highlighted differences between CMJ and MVC.

## Discussion

When a muscle is stretched, both extrafusal and intrafusal muscle fibers are elongated. If the muscle is then passively returned to its initial length, some slack remains in the polar regions of muscle spindles (Proske et al., 1993) causing a reduction of their sensitivity. A voluntary activation of the stretched muscle would then restore the normal sensitivity of muscle spindles due to the mechanism of α and γ motoneurons co-activation. It is, however, unknown, whether following static stretching, muscle spindles are capable of regaining their receptiveness by their own without an α-γ co-activation. Previous studies on muscle stretching seem to suggest that a slow recovery trend is possible (Budini et al., 2017, 2019), but due to methodological limitations, these results are not conclusive. With the present work we demonstrated that after only 30 s of static stretching, tap reflexes are inhibited for at least 10 min and do not show a recovery trend when the respective limb is in a resting position. It could have been anticipated, that when not self-restored due to elastic properties of the intrafusal muscle fibers, tap reflex briskness could have regained due to the activation of the reflex loop itself. It is in fact conceivable that Ia afferents, by activating α motoneurons, would also synapse with γ motoneurons. However, γ motoneuron do not receive monosynaptic excitatory action from Ia afferents (Eldred et al., 1953; Hunt and Paintal, 1958), and this is in line with our results.

Contrary, reflexes were immediately restored after a maximal voluntary contraction of the stretched muscle. This result was expected and can be undoubtedly attributed to α-γ co-activation. The twitch torque was also not significantly different from baseline recordings in direct relation to the muscular activation. However, looking at Figure 3B, although not significant, the twitch torque was on average smaller throughout the 10 min post stretching. This could be attributed to changes in compliance of the muscle-tendon complex which have been demonstrated do be reduced up to at least 5 min following static stretching (Konrad et al., 2019), moreover the maximal voluntary contraction itself might have further transiently increased tendon compliance (Kubo et al., 2001; Kay et al., 2015).

After three counter movement jumps, the tap reflex was still strongly inhibited in all the subjects for about 90 s. This result was unexpected and not easy to clarify with the available data. An explanation could be sought in the specific differences between the two types of voluntary contractions adopted in the present study (dynamic and isometric) and their implication on γ motoneuron activity. It is known that γ motoneurons have a background discharge which can be augmented or inhibited by several structures of the central nervous system (Granit and Kaada, 1953). Hunt and Paintal (1958) demonstrated in animals that muscle nerve volley of one muscle group influences γ motoneuron discharge in another muscle group. More specifically inhibitory projections of quadriceps were observed on γ motoneurons of the ipsilateral gastrocnemius. It could therefore be speculated that during the counter movement jumps, the activation of the knee extensors inhibited the activation of γ motoneurons of the plantar flexors, whilst during isometric contraction the knee extensor muscles were not activated and consequently did not project on plantar flexors γ motoneurons. However, if projections from knee extensors prevented the activation of plantar flexors’ γ motoneurons, then the tap reflex should have not recovered at all, as observed after stretching when this was not followed by a voluntary contraction. On the contrary, the inhibition recovered after 90 s. Neurophysiological responses to stretching, however, do not have such long lasting effects (Budini et al., 2018a, b), similarly inhibitory projections from knee extensors last just few milliseconds (Hunt and Paintal, 1958). Consequently, this result should be attributed to a different mechanism.

By looking at Figure 3C it can be noticed that the inhibition was even more marked than following stretching alone. This could be attributed to the eccentric contraction of plantar flexors during the lowering phase of the counter movement jump and such phase could be considered as a form of dynamic stretching of the calf muscles. Dynamic stretching induces similar effects as static stretching, causing a transient decrease in muscle-tendon stiffness (Konrad et al., 2017). It could therefore be speculated that the dynamic contractions promoted a recovery of muscle spindle sensitivity due to α-γ co-contraction similarly to the isometric contraction, however, due to a transient increase in compliance provoked by dynamic stretching of the triceps surae, the perturbation of the Achilles tendon induced a smaller stretch of the muscle resulting in smaller reflexes. After about 90 s, as the morpho-mechanical property of the muscle-tendon complex returned to normality, also the reflexes returned the same amplitude as at baseline.

### Study Limitations

We cannot exclude that the inhibition observed after the counter movement jumps could be attributed to the methodological requirement of standing up from the dynamometer chair and sit down again for completing the measurement after the exercise. A further investigation should address this aspect by adding a control group that will only stand up for 30 s before sitting down again. Alternatively, renouncing to test an exercise similar to what commonly performed in sport warm-up routines, the dynamic contractions could be performed directly on the dynamometer.

Similarly, we cannot exclude that the strong and transient inhibition observed after the counter movement jumps could be the consequence of the jumps alone and not influenced by the previous 30 s static stretching.

In conclusion, 30 s static stretching induces an inhibition of the tap reflexes that does not recover spontaneously. A static maximal voluntary contraction promotes a quick recovery of reflex sensitivity whilst three dynamic contractions delay the recovery by approximately 90 s. This unexpected effect could be attributed to a transient increase of compliance of the muscle-tendon complex provoked by the eccentric contraction phase of the triceps surae during the counter movement jumps.

## Data Availability Statement

The datasets generated for this study are available on request to the corresponding author.

## Ethics Statement

The studies involving human participants were reviewed and approved by research ethics board of the University of Graz (approval no. GZ. 39/77/63 ex 2013/14). The patients/participants provided their written informed consent to participate in this study.

## Author Contributions

FB, EG, MC, DR, and MT contributed to the conception and design of the work, the drafting the work or revising it critically for important intellectual content, and final approval of the version to be published. FB contributed to the acquisition, analysis, and interpretation of data for the work. All authors contributed to the article and approved the submitted version.

## Conflict of Interest

The authors declare that the research was conducted in the absence of any commercial or financial relationships that could be construed as a potential conflict of interest.
